# Naturalistic psilocybin use is associated with persisting improvements in mental health and wellbeing: results from a prospective, longitudinal survey

**DOI:** 10.3389/fpsyt.2023.1199642

**Published:** 2023-09-19

**Authors:** Sandeep M. Nayak, Hillary Jackson, Nathan D. Sepeda, David S. Mathai, Sara So, Abigail Yaffe, Hadi Zaki, Trey J. Brasher, Matthew X. Lowe, Del R. P. Jolly, Frederick S. Barrett, Roland R. Griffiths, Justin C. Strickland, Matthew W. Johnson, Heather Jackson, Albert Garcia-Romeu

**Affiliations:** ^1^Department of Psychiatry and Behavioral Sciences, Johns Hopkins University School of Medicine, Baltimore, MD, United States; ^2^Center for Psychedelic and Consciousness Research, Johns Hopkins University School of Medicine, Baltimore, MD, United States; ^3^Center for Psychedelic Drug Research and Education, The Ohio State University, Columbus, OH, United States; ^4^Unlimited Sciences, Colorado Springs, CO, United States; ^5^Department of Psychological and Brain Sciences, Johns Hopkins University, Baltimore, MD, United States; ^6^Department of Neuroscience, Johns Hopkins University School of Medicine, Baltimore, MD, United States

**Keywords:** psilocybin, psychedelic, hallucinogen, crowdsourced data, mystical experience

## Abstract

**Introduction:**

The classic psychedelic psilocybin, found in some mushroom species, has received renewed interest in clinical research, showing potential mental health benefits in preliminary trials. Naturalistic use of psilocybin outside of research settings has increased in recent years, though data on the public health impact of such use remain limited.

**Methods:**

This prospective, longitudinal study comprised six sequential automated web-based surveys that collected data from adults planning to take psilocybin outside clinical research: at time of consent, 2 weeks before, the day before, 1–3 days after, 2–4 weeks after, and 2–3 months after psilocybin use.

**Results:**

A sample of 2,833 respondents completed all baseline assessments approximately 2 weeks before psilocybin use, 1,182 completed the 2–4 week post-use survey, and 657 completed the final follow-up survey 2–3 months after psilocybin use. Participants were primarily college-educated White men residing in the United States with a prior history of psychedelic use; mean age = 40 years. Participants primarily used dried psilocybin mushrooms (mean dose = 3.1 grams) for “self-exploration” purposes. Prospective longitudinal data collected before and after a planned psilocybin experience on average showed persisting reductions in anxiety, depression, and alcohol misuse, increased cognitive flexibility, emotion regulation, spiritual wellbeing, and extraversion, and reduced neuroticism and burnout after psilocybin use. However, a minority of participants (11% at 2–4 weeks and 7% at 2–3 months) reported persisting negative effects after psilocybin use (e.g., mood fluctuations, depressive symptoms).

**Discussion:**

Results from this study, the largest prospective survey of naturalistic psilocybin use to date, support the potential for psilocybin to produce lasting improvements in mental health symptoms and general wellbeing.

## Introduction

Psilocybin is a naturally occurring serotonergic psychedelic found in more than 200 species of fungi primarily from the genus *Psilocybe* (1). Archeological artifacts indicate Mesoamerican cultures over a wide geographic area may have consumed psilocybin-containing fungi dating back thousands of years (2, 3). In these preindustrial cultures, evidence suggests psilocybin was used in ritualized contexts for healing, religious rites, and divination (2, 4). Prior to regulatory restrictions in the early 1970s, serotonergic psychedelics such as lysergic acid diethylamide (LSD) and psilocybin were studied extensively as pharmacological agents capable of producing unique psychoactive effects often interpreted as deeply personally meaningful (5, 6). Studies included investigation of possible therapeutic uses of psychedelics to treat a variety of conditions, including alcohol dependence, neurosis, and existential distress (7, 8).

In recent years, psilocybin has reemerged as a topic of interest in clinical research, showing good potential in the treatment of existential distress (9–11), mood (12–16), and substance use disorders (17–19) across a series of small pilot studies and preliminary randomized controlled trials. Beyond its potential for treating psychiatric conditions, research in healthy volunteers suggests psilocybin may hold benefits more generally for psychological wellbeing and enhancement of spiritual practices such as meditation (20–25). With growing cultural interest in psychedelics and concurrent policy initiatives to allow for decriminalized psychedelic use in various jurisdictions, naturalistic use of psilocybin outside of laboratory and research settings has also increased in recent years (26, 27). However, data on the public health impact of naturalistic psilocybin use outside controlled research settings remain limited, fueling concerns about rising psilocybin use and widespread popular media coverage that may not accurately reflect evidence-based evaluation (28).

The current longitudinal online survey study was conducted to gather additional prospective data on contemporary naturalistic psilocybin use and to provide further insight into the patterns and outcomes surrounding that use. Specifically, the study aims were to: (1) characterize respondent demographics and self-reported psilocybin use patterns including factors such as dose of psilocybin and setting where psilocybin was used; (2) prospectively assess self-reported changes in physical and mental health, personality, wellbeing, and psychological functioning from before to after naturalistic psilocybin use; (3) examine relationships between aspects of individual mindset (e.g., State of Surrender) prior to psilocybin use and observed outcomes during (e.g., subjective drug effects) and after dosing (i.e., persisting effects); and (4) to examine relationships between elements of the setting in which psilocybin use occurred (e.g., presence of a sitter) and observed outcomes during and after dosing.

We tested several *a priori* hypotheses that were informed by prior research and clinical observations. First, we hypothesized respondents would exhibit, on average, persisting improvements in mental health, wellbeing, and psychological functioning from before to after psilocybin use, consistent with clinical trial data and cross-sectional surveys. Second, we hypothesized that aspects of individual mindset (i.e., absorption, effects of adverse childhood experiences, State of Surrender) before the experience would be significantly associated with subjective drug effects (i.e., mystical and challenging experiences) and would predict persisting effects (i.e., changes in longitudinal variables). More specifically, we hypothesized that greater absorption and State of Surrender would be associated with greater mystical-type effects (19, 29–31), that greater adverse childhood experiences would be associated with more challenging effects, and that greater mystical-type effects would be associated with greater mental health and wellbeing improvements (9, 22, 32–34). Finally, we hypothesized that presence of a sitter during the experience would be associated with more positive persisting mental health outcomes (35).

## Methods

### Study design

This prospective, longitudinal survey study enrolled English-speaking adults (≥ 18 years old) planning to take psilocybin outside a clinical research setting. The study was comprised of six sequential web-based surveys that were automated through the Qualtrics XM secure online platform. This study was approved by an Institutional Review Board at the Johns Hopkins University School of Medicine. Recruitment advertisements were shared online through social media and via word of mouth. Initial study information was provided on the website, explaining, “The researchers conducting this study do not advocate or promote psilocybin or other drug use. The aim of this research is to sample people whose intent to take psilocybin is already established. This study is designed for individuals who are planning to take psilocybin in a single-dose session and is not designed for a recurring microdosing regimen.” Following an initial informed consent and demographics survey, participants completed 5 surveys with timing relative to the reference psilocybin experience: 2 weeks before, 1 day before, 1 to 3 days after, 2–4 weeks after, and 2–3 months after. Responses were collected from July 22, 2020 to July 14, 2022. At two occasions during data collection, several novel items and measures were added to address new questions of interest regarding effects of psilocybin on insomnia, sexual satisfaction, worldview, shame, guilt, and symptoms of traumatic brain injury. Those data will be reported separately.

#### Survey 1: consent

Participants reviewed a waiver of documentation of informed consent document explaining the study procedures, confirmed inclusion criteria, and provided basic demographic information including age, gender, race/ethnicity, education, and mental health history. Participants also recorded the purpose and intended date of the planned psilocybin experience. An email address was provided to which subsequent surveys and reminders were automatically sent at pre-set times before and after the date of the planned psilocybin use.

#### Survey 2: 2 weeks pre-session and longitudinal assessments

Participants completed a battery of baseline measures in this survey approximately 2 weeks before the planned psilocybin experience. Some measures were collected only once before the dosing session, including a drug use history, the 34-item Tellegen Absorption Scale (TAS) assessing openness to altered states, scored 0–34 with higher scores indicating greater absorption (36), and the 14-item Adverse Childhood Experience (ACE) scale (revised) assessing history of childhood physical and emotional abuse or neglect, scored 0–14 with higher scores indicating greater incidence of adverse childhood experiences (37). Additionally, a series of assessments were administered repeatedly before and after the planned psilocybin experience. Participants completed these longitudinal assessments in this 2 week pre-session survey, and again in the 2–4 week follow-up, and 2–3 month follow-up surveys. These included a modified 20-item Beck Depression Inventory II (BDI-II) to assess depressed mood (excluding an item about current suicidality due to lack of ability to respond adequately to potential imminent risk), scored 0–60 with higher scores indicating greater severity of depressive symptoms (38); the 10-item Alcohol Use Disorders Identification Test (AUDIT) assessing alcohol consumption and related problems, scored 0–40 with higher scores indicating greater alcohol consumption (39); the validated 20-item Short State–Trait Anxiety Inventory (STAI) assessing state (current) and trait (general) anxiety, scored 10–40 for each subscale with greater scores indicating more anxiety (40, 41); the 10-item Emotion Regulation Questionnaire (ERQ) assessing cognitive reappraisal (i.e., ability to view emotional stimuli in a variety of ways, scored 6–42, with higher scores indicating greater use of cognitive reappraisal) and expressive suppression (i.e., tendency to suppress emotional response in a given context, scored 4–28, with higher scores indicating greater use of expressive suppression) as two dimensions of emotion regulation (42); the 12-item Cognitive Flexibility Scale (CFS) assessing self-reported ability to think and behave adaptively, scored from 12 to 72 with higher scores indicating greater cognitive flexibility (43); the 4-item Patient-Reported Outcomes Measurement Information System Global Health (PROMIS-GH) physical health subscale assessing self-reported physical health, scored 4–20 with higher scores indicating greater physical health (44); the 12-item Functional Assessment of Chronic Illness Therapy Spiritual Wellbeing (FACIT-Sp) assessing spiritual wellbeing dimensions of faith, meaning, and peace, scored 0–48 with higher scores indicating greater spiritual wellbeing (45); and the 13-item Copenhagen Burnout Inventory (CBI) assessing personal and work-related burnout and emotional exhaustion, both scored 0–100 with higher scores indicating greater burnout (46). Finally, the 44-item Big Five Inventory (BFI) assessed five major dimensions of personality: Openness, Conscientiousness, Extraversion, Agreeableness, and Neuroticism at 2 weeks prior and 2–3 months after the planned psilocybin experience, scored 1–5, with higher scores indicating greater magnitude of each dimension.

#### Survey 3: day prior to session

Participants were asked what dose they were planning to take, the form of psilocybin (dried, wet, steeped/tea, truffles, etc.), whether a sitter/guide would be present, and that person’s qualifications. Additional data were collected on the purpose of the session, outlook regarding the session, and the 10-item State of Surrender (SOS) scale, which assesses level of psychological surrender or preoccupation before the session, that have previously shown correlations to mystical and challenging subjective effects of psilocybin, respectively (47).

#### Survey 4: 1–3 days after session

Participants were asked what form of psilocybin they took, an estimated dosage, and questions about the setting in which the experience took place: with whom they took psilocybin (e.g., alone, with friends, with a sober sitter) and where (e.g., home, outdoors in nature, a religious or spiritual setting, a festival). In addition, participants completed measures of the subjective qualities of the psychedelic experience. The 30-item Mystical Experience Questionnaire (MEQ30) was used to assess the degree of mystical-type qualities of the psychedelic experience (i.e., unitive experience; positive mood; transcending space or time; ineffability), with scores scaled from 0 to 1 and scores ≥ 60% of the maximum score on each of the 4 subscales indicating a “complete mystical experience” (48). The 26-item Challenging Experience Questionnaire (CEQ) assessed a variety of difficult experiences that could arise during the psilocybin session, comprised of seven factors: grief, fear, death, insanity, isolation, physical distress, and paranoia (49). CEQ scores were scaled from 0 to 1 with higher scores indicating a greater degree of challenging experience.

#### Surveys 5 and 6: follow-ups at 2–4 weeks and 2–3 months post-psilocybin

In surveys 5 and 6, completed approximately 2–4 weeks and 2–3 months after the psilocybin experience, respectively, participants were asked to rate the meaningfulness, insightfulness, and spiritual significance of the experience. Items asked: “How personally meaningful/psychologically insightful/spiritually significant was your psilocybin experience and your contemplation of that experience?” Responses ranged across eight options from “No more than routine, everyday experiences” to “The single most meaningful/insightful/spiritually significant experience of my life.” As noted above, the BFI was re-administered in survey 6 at 2–3 months after the psilocybin experience. Otherwise, all longitudinal measures (i.e., BDI-II, AUDIT, STAI, ERQ, CFS, PROMIS-GH, FACIT-Sp, CBI) were re-administered in both of these surveys.

#### Data analysis

Descriptive statistics including means, SD, and ranges were performed for demographic variables. For longitudinal measures, linear mixed models using the *lme4* package (50) in *R* were used with the following covariates: baseline score of each measure, time (2-weeks prior to session, 2–4 weeks after the session, or 2–3 months after), age, sex, race (White or non-White), education level, the presence of a sitter, dose of psilocybin mushroom in dried grams, whether the participant had ever taken a psychedelic before, ACE score, MEQ30 score, CEQ score, State of Surrender score, and TAS score. MEQ30, CEQ, State of Surrender, and TAS also included interactions with time, and models included random intercepts for participant to account for repeated measures. Eleven such models were used for the following outcomes, which were assessed 2-weeks prior to the session, 2–4 weeks after and 2–3 months after: BDI, AUDIT, STAI-Trait, STAI-State, CFS, PROMIS-GH physical health, FACIT-Sp, the expressive suppression and cognitive reappraisal subscales of the ERQ, and the personal and work burnout subscales of the CBI. Model results for individual regression parameters are reported as beta regression coefficients (β) and *p*-values. *p*-values for categorical demographic variables with more than 2 levels (Education and Sex) were calculated using type II Wald Chi-square tests using the Anova function of the *car* package (51). This is an omnibus test performed on the regression model that allows for a single *p*-value for categorical variables with multiple levels.

Effects on longitudinal measures are presented as covariate-adjusted mean differences between timepoints—both unstandardized and standardized mean differences (SMD). These SMDs are interpretable as covariate-adjusted Cohen’s *d*’s. SMDs reflecting changes between timepoints were calculated as follows: first, linear mixed-models were performed with the outcome variables Z-scored. Then, covariate-adjusted contrasts were calculated between each follow-up timepoint and baseline using the emmeans function of the *emmeans* package (52).

For the five dimensions of the Big Five Inventory, simple linear models were performed with time as the predictor of interest and baseline score as a covariate. Additionally, correlations between adverse childhood experience (ACE) scores and subjective drug effects (MEQ30 and CEQ) were calculated using Kendall correlations. Similarly, correlations between Tellegen Absorption Scale (TAS) and State of Surrender (SOS) scores were performed with subjective drug effects measures (MEQ30 and CEQ) using Pearson correlations. Eleven mixed models for longitudinal variables, 5 linear models total for the Big Five Inventory, and 6 correlations total 22 statistical significance tests. The Šidák correction was used with an unadjusted alpha of 0.05 and 22 tests for a corrected alpha threshold of 0.0023.

Two additional exploratory analyses were included to examine the effects of concurrent antidepressant use on acute subjective effects of psilocybin. Means and standard deviations for MEQ30 and CEQ were calculated for those endorsing antidepressant use of any kind (this was not differentiated by antidepressant drug class) during the psilocybin experience. These scores were compared with MEQ30 and CEQ ratings of individuals who were not taking an antidepressant during their psilocybin experience using independent samples t-tests. As exploratory measures, these were not included in the calculation of the Šidák correction above.

## Results

### Participant demographics

Sample sizes for each of the surveys were *N* = 8,006 (Survey 1; consent), *N* = 2,833 (Survey 2; 2 weeks pre), *N* = 1,802 (Survey 3; 1 day pre), *N* = 1,551 (Survey 4; 1–3 days post), *N* = 1,182 (Survey 5; 2–4 weeks post), *N* = 657 (Survey 6; 2–3 months post). Demographics across timepoints are displayed in Table 1. Participants were majority White (81–87%), male (54–59%), residing in the United States (73–83%), and had previous experience using psychedelics (86–87%). Mean age was about 40 years old. More than half the sample (54–66%) held a bachelor’s level or higher degree. Participants reported using psilocybin 16–17 times on average prior to enrolling in the study.

**Table 1:** Participant demographics across timepoints.

	Informed consent	2-weeks pre	1-day pre	1-3-days post	2–4 weeks post	2–3 months post
N	8,006	2,833	1802	1,551	1,182	657
Current age, mean (SD) [range]	38.9 (13.1) [18, 89]	39.8 (13.1) [18, 89]	39.7 (12.7) [18, 81]	40.1 (12.7) [18, 78]	40.4 (13) [18, 78]	41 (13.2) [18, 89]
Male sex (%)	4,334 (54.1)	1,531 (54)	968 (53.7)	830 (53.5)	635 (53.7)	388 (59.1)
Race						
White	6,464 (80.7)	2,306 (81.4)	1,492 (82.8)	1,297 (83.6)	995 (84.2)	568 (86.5)
Asian	257 (3.2)	96 (3.4)	61 (3.4)	53 (3.4)	39 (3.3)	18 (2.7)
Black	143 (1.8)	45 (1.6)	25 (1.4)	18 (1.2)	8 (0.7)	4 (0.6)
Native American	101 (1.3)	28 (1)	14 (0.8)	11 (0.7)	7 (0.6)	3 (0.5)
Other	1,025 (12.8)	353 (12.5)	206 (11.4)	168 (10.8)	130 (11)	63 (9.6)
Hispanic (%)	930 (11.6)	302 (10.7)	186 (10.3)	149 (9.6)	116 (9.8)	67 (10.2)
Marital status						
Married	2,518 (31.5)	959 (33.9)	651 (36.1)	593 (38.2)	454 (38.4)	252 (38.4)
Single	2,523 (31.5)	844 (29.8)	507 (28.1)	412 (26.6)	315 (26.6)	180 (27.4)
In a committed relationship (not married)	1909 (23.8)	674 (23.8)	424 (23.5)	365 (23.5)	270 (22.8)	154 (23.4)
Divorced	762 (9.5)	250 (8.8)	156 (8.7)	131 (8.4)	104 (8.8)	52 (7.9)
Separated	204 (2.5)	71 (2.5)	42 (2.3)	33 (2.1)	23 (1.9)	13 (2)
Widowed	90 (1.1)	35 (1.2)	22 (1.2)	17 (1.1)	16 (1.4)	6 (0.9)
Country						
United States	6,655 (83.1)	2,234 (78.9)	1,420 (78.8)	1,206 (77.8)	902 (76.3)	477 (72.6)
Canada	323 (4)	161 (5.7)	108 (6)	94 (6.1)	74 (6.3)	47 (7.2)
United Kingdom	164 (2)	65 (2.3)	42 (2.3)	39 (2.5)	34 (2.9)	20 (3)
Germany	56 (0.7)	27 (1)	16 (0.9)	17 (1.1)	16 (1.4)	12 (1.8)
Netherlands	44 (0.5)	20 (0.7)	16 (0.9)	17 (1.1)	13 (1.1)	7 (1.1)
Mexico	44 (0.5)	22 (0.8)	13 (0.7)	11 (0.7)	8 (0.7)	6 (0.9)
Education						
High school/GED or less	121 (1.5)	38 (1.3)	23 (1.3)	18 (1.2)	17 (1.4)	11 (1.7)
Some college, no degree	1770 (22.1)	546 (19.3)	327 (18.1)	279 (18)	198 (16.8)	90 (13.7)
Trade school/Associates degree	582 (7.3)	204 (7.2)	139 (7.7)	111 (7.2)	74 (6.3)	43 (6.5)
Bachelor’s degree	2,429 (30.3)	897 (31.7)	576 (32)	497 (32)	388 (32.8)	221 (33.6)
Master’s degree	1,360 (17)	532 (18.8)	356 (19.8)	318 (20.5)	254 (21.5)	146 (22.2)
Advanced professional degree (e.g., Ph.D./MD)	524 (6.5)	227 (8)	148 (8.2)	141 (9.1)	112 (9.5)	66 (10)
Drug use history						
No psychedelic experience prior to study, n (%)	n/a	410 (14.5)	257 (14.3)	223 (14.4)	172 (14.6)	86 (13.1)
Number prior psychedelic experiences (mean, SD) [range]	n/a	29.2 (44.3) [0, 400]	28.8 (43.8) [0, 400]	28.1 (43.3) [0, 400]	27.1 (43.3) [0, 400]	27.4 (44.6) [0, 400]
Number prior psilocybin experiences (mean, SD) [range]	n/a	17.1 (21.8) [0, 100]	16.9 (21.5) [0, 100]	16.5 (21.3) [0, 100]	15.7 (20.7) [0, 100]	15.5 (19.5) [0, 100]
Number prior LSD experiences (mean, SD) [range]	n/a	12.4 (21.9) [0, 100]	12.1 (21.7) [0, 100]	11.8 (21.4) [0, 100]	11.3 (20.8) [0, 100]	10.7 (20.7) [0, 100]
Number prior ayahuasca experiences (mean, SD) [range]	n/a	1.1 (6.1) [0, 100]	1.1 (6.4) [0, 100]	1.2 (6.7) [0, 100]	1.3 (7.4) [0, 100]	1.7 (8.6) [0, 100]
Number prior mescaline experiences (mean, SD) [range]	n/a	1.3 (6.2) [0, 100]	1.3 (6.4) [0, 100]	1.2 (6) [0, 100]	1.2 (6.3) [0, 100]	1.3 (6.9) [0, 100]
Number prior DMT experiences (mean, SD) [range]	n/a	2.3 (9.2) [0, 100]	2.3 (9.4) [0, 100]	2.3 (9.4) [0, 100]	2.3 (9.8) [0, 100]	2.4 (10.8) [0, 100]
Current health conditions						
Anxiety disorder, n (%)	2,747 (34.3)	905 (31.9)	572 (31.7)	482 (31.1)	355 (30)	172 (26.2)
Eating disorder, n (%)	190 (2.4)	65 (2.3)	45 (2.5)	36 (2.3)	26 (2.2)	12 (1.8)
Impulse control disorder, n (%)	89 (1.1)	33 (1.2)	24 (1.3)	19 (1.2)	10 (0.8)	5 (0.8)
Mood disorder, n (%)	2,380 (29.7)	811 (28.6)	524 (29.1)	444 (28.6)	339 (28.7)	172 (26.2)
Personality disorder, n (%)	291 (3.6)	94 (3.3)	55 (3.1)	47 (3)	32 (2.7)	17 (2.6)
Chronic pain, n (%)	882 (11)	272 (9.6)	175 (9.7)	145 (9.3)	111 (9.4)	64 (9.7)
Psychotic disorder, n (%)	29 (0.4)	7 (0.2)	4 (0.2)	3 (0.2)	1 (0.1)	0 (0)
Substance use disorder, n (%)	463 (5.8)	138 (4.9)	76 (4.2)	57 (3.7)	37 (3.1)	16 (2.4)

### Intention

In survey 3 (*n* = 1,802), completed immediately before the psilocybin experience, participants reported (non-exclusively) self-exploration (*n* = 1,461; 81.1%), mental health (*n* = 1,284; 71.3%), therapy (*n* = 863; 47.9%), creativity (*n* = 787; 43.7%), recreation (*n* = 680; 37.7%), productivity (*n* = 403; 22.4%), and physical health (*n* = 258; 14.3%) as their motivations for the experience. About three quarters (*n* = 1,336; 74.1%) of respondents reported setting a specific intention for the experience. For example, one participant stated, “I intend to fully reconnect with myself and my purpose after my cancer surgery and dissolve any obstacles, blocks, or fears that are preventing me from moving forward.” Another wrote, “I want to feel connected to the world and people around me.”

### Setting

In survey 4 (*n* = 1,551), completed 1 to 3 days after the psilocybin experience, participants reported primarily using psilocybin alone (*n* = 667; 43.0%), with friends who were also using psilocybin (*n* = 399; 25.7%), or with a sober friend serving as a sitter (*n* = 255; 16.4%). Some also reported using psilocybin with a shaman or guide (*n* = 23; 1.5%), with a guided group (*n* = 22; 1.4%), or with a therapist (*n* = 17; 1.1%). Most participants used psilocybin at home (*n* = 1,081; 69.7%) or outdoors in nature (*n* = 245; 15.8%). Smaller proportions reported using psilocybin in a religious or spiritual setting (*n* = 39; 2.5%), at a concert or festival (*n* = 18; 1.2%); at a party (*n* = 11; 0.7%), or in another public place (e.g., mall, movie theater; *n* = 11; 0.7%). The median time of dosing was 4 pm, and the modal time was 11 am.

### Dosage, form, and other substances

*Psilocybe cubensis* was the most commonly noted mushroom species in survey 4, used by 675 (43.5%) participants, while 250 (16.1%) indicated another mushroom species, and the remaining 626 (40.4%) were unsure of the type of mushroom being used. Participants largely reported taking dried whole mushrooms (*n* = 655; 42.2%), dried ground mushrooms (*n* = 296; 19.3%), mushrooms steeped in tea (*n* = 241; 15.5%), and mushroom-infused edibles (e.g., chocolates; *n* = 99; 6.4%). A minority (*n* = 193; 12.4%) reported taking more than one dose of psilocybin during the reference experience, with most of these (*n* = 151) taking 2 total doses, 36 taking 3 doses, and 6 taking 4 or more doses during the session. Excluding outliers (i.e., <0.2 g; *n* = 27; 1.7%; and > 15 g; *n* = 30; 1.9%), among individuals who reported dosage in grams (*n* = 1,501; 96.8%), the average (SD) initial dose was 3.1 g (2.3). During the experience, the most common substances used in conjunction with psilocybin were cannabis (*n* = 479; 30.9%), caffeine (*n* = 229; 14.8%), and alcohol (*n* = 179; 11.5%). Additionally, 72 people (4.6%) reported using psilocybin while on an antidepressant. Only 35 people (2.3%) reported taking another psychedelic during their psilocybin experience.

### Subjective effects

Among individuals completing survey 4, the mean (SD) total MEQ30 score was 0.5 (0.25), with 335 (21.6%) meeting *a priori* criteria for a “complete mystical experience.” The mean (SD) total CEQ was 0.14 (0.14). MEQ30 score was a significant predictor of changes in multiple longitudinal variables including decreased depression, personal burnout, work burnout, and state anxiety, as well as increased cognitive flexibility and spiritual wellbeing (Table 2). CEQ score was not found to predict linear changes in any longitudinal measures.

**Table 2:** Beta-coefficients (unstandardized) and *p*-values for linear mixed models between longitudinal measures and covariates.

	BDI	AUDIT	STAI state	STAI trait	ERQ (cognitive reappraisal)	ERQ (expressive suppression)	Cognitive flexibility	FACIT-Sp	Burnout (Personal)	Burnout (Work)	PROMIS-GH physical health
Intercept	**9.82 (<0.001)**	0.58 (0.34)	**7.2 (<0.001)**	4.45 (0.01)	**1.06 (<0.001)**	0.82 (0.002)	**15.25 (<0.001)**	0.94 (0.65)	**8.94 (<0.001)**	7.74 (0.01)	**4.13 (<0.001)**
Baseline score	**0.63 (<0.001)**	**0.9 (<0.001)**	**0.74 (<0.001)**	**0.8 (<0.001)**	**0.69 (<0.001)**	**0.81 (<0.001)**	**0.67 (<0.001)**	**7.41 (<0.001)**	**0.83 (<0.001)**	**0.85 (<0.001)**	**0.72 (<0.001)**
Time 2–4 week	**−16.9 (<0.001)**	−0.35 (0.58)	−2.84 (0.19)	−2.25 (0.23)	**0.93 (<0.001)**	0.19 (0.49)	3.77 (0.04)	4.78 (0.03)	−7.55 (0.005)	5.06 (0.12)	0.24 (0.59)
Time 2–3 month	**−9.26 (<0.001)**	−0.74 (0.24)	−0.4 (0.85)	−1.75 (0.35)	0.62 (0.03)	0.12 (0.68)	2.19 (0.23)	**0.74 (<0.001)**	−8.24 (0.01)	1.72 (0.67)	0.19 (0.66)
MEQ-30	0.85 (0.5)	0.07 (0.84)	−0.49 (0.65)	−0.42 (0.64)	0.1 (0.44)	0.01 (0.92)	−0.08 (0.93)	1.02 (0.36)	−0.53 (0.66)	0.78 (0.58)	0.05 (0.81)
CEQ	2.16 (0.32)	0.24 (0.67)	2.13 (0.26)	2.07 (0.2)	−0.16 (0.5)	0.01 (0.98)	−1.88 (0.24)	−1.91 (0.33)	4.12 (0.04)	1.99 (0.38)	0.24 (0.51)
Age	0.02 (0.33)	0 (0.24)	−0.02 (0.2)	−0.01 (0.44)	0 (0.28)	0 (0.93)	−0.01 (0.33)	−0.02 (0.27)	0.02 (0.16)	−0.02 (0.33)	−0.01 (0.02)
Sex*	(0.08)	(0.78)	(0.03)	(0.03)	(0.14)	(0.32)	(0.31)	(0.12)	(0.02)	(0.004)	(0.13)
White	−0.72 (0.17)	0.24 (0.11)	0.59 (0.22)	0.61 (0.14)	0.01 (0.9)	−0.09 (0.14)	0.66 (0.11)	−0.23 (0.66)	−0.45 (0.41)	−0.19 (0.77)	−0.1 (0.28)
Education*	(0.67)	(0.6)	(0.94)	(0.7)	(0.57)	(0.8)	(0.15)	(0.007)	(0.41)	(0.42)	(0.23)
TAS	−0.05 (0.22)	−0.01 (0.26)	0 (1)	0 (0.93)	0.01 (0.004)	0 (0.27)	0.05 (0.08)	0.11 (0.002)	0 (0.9)	0.01 (0.84)	0 (0.56)
ACE	0.34 (0.004)	−0.01 (0.65)	0.24 (0.02)	0.23 (0.01)	−0.01 (0.3)	0.01 (0.47)	−0.02 (0.82)	−0.19 (0.08)	**0.37 (0.001)**	0.17 (0.22)	−0.04 (0.05)
Sitter	0.19 (0.62)	0.21 (0.05)	−0.06 (0.86)	0.32 (0.3)	−0.03 (0.46)	−0.01 (0.8)	0.18 (0.54)	0.06 (0.86)	−0.11 (0.8)	1.21 (0.02)	0.08 (0.23)
First psychedelic experience	0.44 (0.43)	−0.04 (0.78)	0.56 (0.25)	0.33 (0.44)	−0.11 (0.06)	0.12 (0.07)	−0.19 (0.64)	−0.32 (0.54)	1.15 (0.05)	0.26 (0.71)	−0.08 (0.36)
Dose (grams)	0.05 (0.48)	**−0.07 (<0.001)**	0.01 (0.82)	0.04 (0.45)	0 (0.65)	0 (0.67)	0.02 (0.65)	−0.05 (0.48)	−0.06 (0.44)	−0.03 (0.74)	0 (0.94)
SOS	**−2.07 (0.002)**	−0.04 (0.82)	−1.13 (0.05)	−0.87 (0.08)	0.15 (0.04)	−0.05 (0.53)	1.17 (0.02)	**2.05 (<0.001)**	−1.65 (0.01)	−0.79 (0.29)	0.06 (0.56)
MEQ-30 × Time 2–4 week	**−7.49 (<0.001)**	−0.25 (0.52)	**−4.31 (0.001)**	−2.61 (0.02)	0.42 (0.02)	−0.47 (0.01)	**6.46 (<0.001)**	**8.87 (<0.001)**	**−13.39 (<0.001)**	−6.26 (0.003)	−0.23 (0.39)
MEQ-30 × Time 2–3 month	**−9.66 (<0.001)**	−0.47 (0.23)	**−4.17 (0.002)**	**−3.67 (0.001)**	**0.53 (0.002)**	−0.31 (0.07)	**5.39 (<0.001)**	**7.27 (<0.001)**	**−12.59 (<0.001)**	**−10.33 (<0.001)**	0.36 (0.18)
CEQ × Time 2–4 week	1.45 (0.62)	−0.01 (0.99)	−1.88 (0.43)	−2.45 (0.23)	−0.15 (0.63)	0.2 (0.51)	−0.07 (0.97)	−1.51 (0.53)	1.04 (0.71)	−2.47 (0.46)	0.36 (0.45)
CEQ × Time 2–3 month	−1.17 (0.7)	0.31 (0.66)	−0.73 (0.76)	−0.57 (0.78)	−0.12 (0.68)	0.05 (0.86)	1.56 (0.43)	0.73 (0.76)	0.85 (0.81)	0.32 (0.94)	−0.66 (0.17)
TAS × Time 2–4 week	0.11 (0.04)	0.01 (0.54)	0.03 (0.47)	0.05 (0.22)	**−0.02 (<0.001)**	**0.02 (0.002)**	−0.07 (0.04)	**−0.19 (<0.001)**	0.13 (0.02)	0.02 (0.79)	−0.01 (0.38)
TAS × Time 2–3 month	0.13 (0.02)	0.01 (0.47)	−0.03 (0.56)	0.01 (0.85)	**−0.02** **(<0.001)**	0.02 (0.01)	−0.07 (0.07)	−0.13 (0.002)	0.09 (0.18)	−0.05 (0.57)	−0.01 (0.35)
ACE × Time 2–4 week	**−0.8 (<0.001)**	0 (0.92)	−0.24 (0.07)	−0.3 (0.01)	0 (0.92)	−0.01 (0.49)	0.07 (0.54)	**0.49 (<0.001)**	**−0.91 (<0.001)**	0.07 (0.7)	0.01 (0.74)
ACE × Time 2–3 month	**−0.56 (<0.001)**	0.01 (0.8)	−0.16 (0.21)	−0.23 (0.04)	0.03 (0.07)	−0.03 (0.12)	0.01 (0.91)	0.4 (0.002)	**−0.99 (<0.001)**	0.1 (0.68)	−0.01 (0.8)
SOS × Time 2–4 week	**4.24 (<0.001)**	0 (0.98)	1.09 (0.12)	0.58 (0.33)	−0.16 (0.08)	−0.13 (0.16)	−1.34 (0.02)	−1.71 (0.02)	2.38 (0.01)	−1.14 (0.29)	−0.02 (0.91)
SOS × Time 2–3 month	2.29 (0.01)	0.1 (0.64)	0.57 (0.42)	0.7 (0.25)	−0.08 (0.4)	−0.1 (0.27)	−0.73 (0.21)	−0.91 (0.2)	2.57 (0.02)	0.16 (0.91)	0 (0.98)

ACE, Adverse Childhood Experiences Scale (revised); AUDIT, Alcohol Use Disorders Identification Test; BDI-II, Beck Depression Inventory-II; CEQ, Challenging Experience Questionnaire; ERQ, Emotional Regulation Questionnaire; FACIT-Sp, Functional Assessment of Chronic Illness Therapy-Spiritual Wellbeing Scale; MEQ30, Mystical Experience Questionnaire-30; PROMIS-GH, Patient Reported Outcomes Measurement Information System (PROMIS) Global Health; SOS, State of Surrender; STAI, State Trait Anxiety Inventory; TAS, Tellegen Absorption Scale. Bolded values are significant at *p* < 0.0023.

*p-values for sex and education are presented as the result of a Wald type II Chi-square omnibus test across all levels of those variables. Thus, regression coefficients are not reported for each level of these variables.

### Negative effects

On survey 4, six respondents (0.4%) reported seeking medical care, and 52 (3.4%) reported seeking psychological care during or in the 1–3 days after the experience. These ranged from mild (e.g., “headache pill, aspirin”) to more severe adverse events, e.g., “I ended up fainting because I lost my self-identity and panicked. I did not know who anyone was or who I was.” Another participant wrote, “I went to the ER for suicidal ideation.” Most incidents of seeking psychological support described calling a friend or family member, or speaking to a counselor or therapist after the experience for integration purposes, and did not represent acute adverse events. Regarding persisting negative effects, 7–11% of participants reported persisting negative effects across follow-up timepoints (Table 3). The most commonly reported persisting negative effects after psilocybin use were mood fluctuations (*n* = 55; 4.7% at 2–4 weeks, and *n* = 20; 3.0% at 2–3 months) and depressive notions (*n* = 37; 3.1% at 2–4 weeks, and *n* = 11; 1.7% at 2–3 months).

**Table 3:** Retrospective ratings on psilocybin experience and subsequent use (Surveys 5 and 6).

	2–4 weeks, mean (SD)	2–3 months, mean (SD)
How personally meaningful was your psilocybin experience and your contemplation of that experience?^1^	4.4 (1.8)	4.5 (1.8)
How spiritually significant was your psilocybin experience and your contemplation of that experience?^1^	4.0 (2.2)	4.3 (2.1)
How personally psychologically insightful was your psilocybin experience and your contemplation of that experience?^1^	4.4 (2.0)	4.5 (2.0)
How psychologically challenging was the most psychologically challenging portion of the psilocybin experience?^1^	3.2 (2.0)	3.3 (2.0)
Do you believe that the psilocybin experience and your contemplation of that experience has led to long-term and persisting changes in your current sense of personal wellbeing or life satisfaction?^1^	2.4 (1.2)	2.2 (1.0)
Have you experienced any persisting negative effects from your psilocybin experience, which lasted beyond the duration of the drug’s effects?^2^	2–4 weeks, n (%)	2–3 months, n (%)
None	1,055 (89.3)	609 (92.7)
Mood fluctuations	55 (4.7)	20 (3.0)
Loneliness	28 (2.4)	6 (0.9)
Lowered motivation	24 (2.0)	10 (1.5)
Depressive notions	37 (3.1)	11 (1.7)
Have you experienced any notable behavioral changes since this psilocybin session?	2–4 weeks, n (%)	2–3 months, n (%)
Reduced or stopped using other drugs	172 (14.6)	121 (18.4)
Started using other drugs more often/heavily	9 (0.8)	7 (1.1)
Reduced craving or use of alcohol	259 (21.9)	162 (24.7)
Increased craving or use of alcohol	7 (0.6)	3 (0.5)
Improved diet/nutrition	285 (24.1)	182 (27.7)
Worsened diet/nutrition	16 (1.4)	4 (0.6)
Increased physical activity/exercise	322 (27.2)	212 (32.3)
Decreased physical activity/exercise	24 (2.0)	10 (1.5)
Improved relationships with others	589 (49.8)	332 (50.5)
Worsened relationships with others	19 (1.6)	10 (1.5)
Improvements in career/work life	313 (26.5)	208 (31.7)
Worsening of career/work life	16 (1.4)	10 (1.5)
None of these	270 (22.8)	149 (22.7)
Which of the following best describes your opinion about the personal use of psilocybin, when taken in the environment you chose for the session?	2–4 weeks, n (%)	2–3 months, n (%)
Extremely negative	0 (0)	0 (0)
Very negative	2 (0.2)	0 (0)
Negative	19 (1.6)	9 (1.4)
Neutral	77 (6.5)	33 (5.0)
Beneficial	269 (22.8)	141 (21.5)
Very beneficial	316 (26.7)	186 (28.3)
Extremely beneficial	499 (42.2)	288 (43.8)
Have you consumed psilocybin mushrooms again since your session?, n (%)	2–4 weeks	2–3 months
Yes	306 (25.9)	331 (50.4)
No	876 (74.1)	326 (49.6)
Number of times psilocybin used again since reference experience, Mean (SD)	0.7 (1.8)	1.8 (3.3)

^1^Ratings provided on the following 8-point scale: No more than routine, everyday personally meaningful/spiritually significant/psychologically insightful/challenging experiences =1. Similar to experiences that occur on average once or more a week = 2. Similar to experiences that occur on average once a month = 3. Similar to experiences that occur on average once a year = 4. Similar to experiences that occur on average once every 5 years = 5. Among the 10 most personally meaningful/spiritually significant/psychologically insightful/challenging experiences of my life = 6. Among the 5 most personally meaningful/spiritually significant/psychologically insightful/challenging experiences of my life = 7. The single most personally meaningful/spiritually significant/psychologically insightful/challenging experience of my life = 8.

^2^Ratings provided on the following 8-point scale: Strong positive change that I consider desirable = 1. Moderate positive change that I consider desirable = 2. Slight positive change that I consider desirable = 3. No change = 4. Slight negative change that I consider undesirable = 5. Moderate negative change that I consider undesirable = 6. Strong negative change that I consider undesirable = 7.

### Longitudinal measures

#### Mood

**Figure 1:**
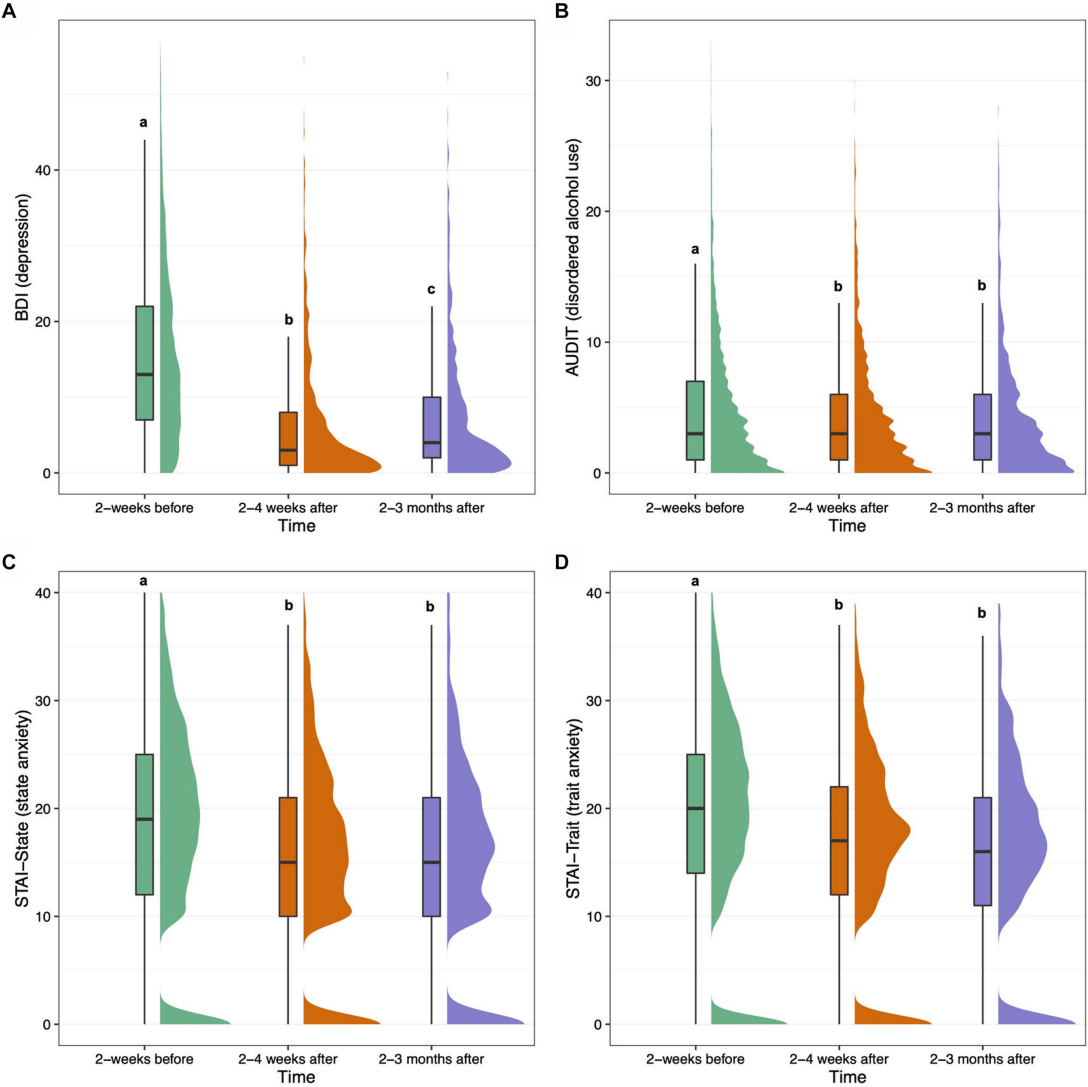
Boxplots and density plots for the following measures over time: modified BDI-II depression **(A)**, AUDIT alcohol use **(B)**, short STAI state anxiety **(C)**, and short STAI trait anxiety **(D)**. Boxes show the interquartile range of responses with the median at the solid horizontal line. Whiskers above and below boxes extend 1.5 times the interquartile range, and density plots show distribution of responses. Values not sharing a common letter above whiskers are significantly different (*p* < 0.0023). Graphs show plotted raw data from all participants with available data at each timepoint.

Modified BDI-II mean (SD) total scores were 15.3 (11.2) for survey 2, with 41.5% (*n* = 1,177) of respondents meeting criteria for some form of depression before their psilocybin experience (i.e., score > 13). For surveys 5 and 6, modified BDI-II mean (SD) total scores were 6.4 (8.3) and 7.5 (8.9), respectively, with 12.6% of respondents (*n* = 149) meeting depression criteria for survey 5, and 14.5% (*n* = 95) meeting depression criteria for survey 6. The adjusted effect size (SMD [95% CI]) of BDI at 2–4 weeks was −0.71 [−0.79, −0.63] (*p* < 0.001), and − 0.58 [−0.66, −0.5] (*p* < 0.001) at 2–3 months, showing significantly decreased depression from baseline to both follow-ups (Table 4). Covariate-adjusted contrasts between each timepoint for modified BDI-II and other longitudinal measures are shown in Figure 1. The following covariates were significant: main effect of time at both timepoints (both negative), baseline BDI, main effect of State of Surrender (negative), the interaction of time at 2–4 weeks and State of Surrender score (positive), and the interaction of time at both timepoints and MEQ30 score (negative) and ACE score (negative). Therefore, depression scores decreased over time relative to baseline, and they decreased to a greater degree the higher the MEQ30 and ACE scores, and decreased to a lesser degree the higher the State of Surrender score. Higher State of Surrender was also associated with lower depression scores overall. Regression coefficients and *p*-values for linear mixed models are displayed in Table 2.

**Table 4:** Covariate-adjusted effect sizes (SMD) of differences between baseline and follow-up longitudinal measures.

	2–4 weeks		2–3 months	
	Mean [95% CI]	*p*	Mean [95% CI]	*p*
BDI-II	−0.71 [−0.79, −0.63]	<0.001	−0.58 [−0.66, −0.5]	<0.001
AUDIT	−0.06 [−0.10, −0.02]	<0.001	−0.09 [−0.13, −0.05]	<0.001
Burnout (personal)	−0.33 [−0.37, −0.29]	<0.001	−0.36 [−0.42, −0.31]	<0.001
Burnout (work)	−0.07 [−0.12, −0.01]	0.01	−0.19 [−0.25, −0.12]	<0.001
Cognitive flexibility	0.23 [0.15, 0.31]	<0.001	0.22 [0.15, 0.30]	<0.001
ERQ (cognitive reappraisal)	0.19 [0.11, 0.27]	<0.001	0.24 [0.16, 0.32]	<0.001
ERQ (expressive suppression)	−0.06 [−0.13, 0.01]	0.11	−0.07 [−0.14, −0.01]	0.03
FACIT-Sp	0.36 [0.29, 0.42]	<0.001	0.38 [0.31, 0.44]	<0.001
Short STAI state	−0.21 [−0.28, −0.13]	<0.001	−0.19 [−0.26, −0.12]	<0.001
Short STAI trait	−0.21 [−0.27, −0.15]	<0.001	−0.21 [−0.27, −0.15]	<0.001
PROMIS-GH physical health	−0.01 [−0.09, 0.07]	0.99	0.06 [−0.02, 0.14]	0.17

BDI-II, Beck Depression Inventory-II; AUDIT, Alcohol Use Disorders Identification Test; ERQ, Emotional Regulation Questionnaire; FACIT-Sp, Functional Assessment of Chronic Illness Therapy-Spiritual Wellbeing Scale; STAI, State Trait Anxiety Inventory; PROMIS-GH, Patient Reported Outcomes Measurement Information System (PROMIS) Global Health.

#### Alcohol use

The mean (SD) score for the AUDIT on survey 2 was 4.8 (5.4), with 16.3% (*n* = 463) of respondents meeting criteria for risky drinking (i.e., score > 7), and 5.9% (*n* = 167) meeting criteria for probable alcohol dependence (i.e., score > 14). For surveys 5 and 6, mean (SD) AUDIT scores were 4.2 (4.7) and 3.9 (4.5), respectively. At survey 5, 13.6% (*n* = 161) of respondents met criteria for risky drinking and 4.8% (*n* = 57) met criteria for probable alcohol dependence. At survey 6, 10.8% (*n* = 71) of respondents met criteria for risky drinking and 4.3% (*n* = 28) met criteria for probable alcohol dependence. The effect size (SMD [95% CI]) of AUDIT at 2–4 weeks was −0.06 [−0.10, −0.02] (*p* < 0.001), and −0.09 [−0.13, −0.05] (*p* < 0.001) at 2–3 months, showing significantly decreased alcohol misuse from baseline to both follow-ups with small effect sizes. The following covariates were significant: baseline score and total dose of psilocybin taken (negative). Therefore, people with lower AUDIT scores overall tended to take higher doses of psilocybin.

#### Anxiety

Short STAI state and trait anxiety mean (SD) total scores were 21.1 (6.9) and 22.0 (6.4) for survey 2, respectively. For survey 2, 28.8% (*n* = 817) of respondents met criteria for high-risk state anxiety (i.e., score > 23), and 32.7% (*n* = 926) met criteria for high-risk trait anxiety (i.e., score > 23). For survey 5, short STAI state and trait anxiety mean (SD) total scores were 18.3 (6.4) and 19.6 (5.9), respectively. For survey 5, 16.2% (*n* = 191) of respondents met criteria for high-risk state anxiety, and 19.5% (*n* = 231) met criteria for high-risk trait anxiety. For survey 6, short STAI state and trait anxiety mean (SD) total scores were 18.3 (6.4) and 19.2 (5.8), respectively. For survey 6, 15.1% (*n* = 99) of respondents met criteria for high-risk state anxiety, and 17.2% (*n* = 113) met criteria for high-risk trait anxiety. The effect size (SMD [95% CI]) of STAI-State at 2–4 weeks was −0.21 [−0.28, −0.13] (*p* < 0.001), and − 0.19 [−0.26, −0.12] (*p* < 0.001) at 2–3 months, showing significantly decreased state anxiety from baseline to both follow-ups. The following covariates were significant: baseline score and the interaction of time and MEQ30 at both timepoints (negative). Therefore, state anxiety scores reduced more over time the higher the MEQ30 score. The effect size (SMD [95% CI]) of STAI-Trait at 2–4 weeks was −0.21 [−0.27, −0.15] (*p* < 0.001), and − 0.21 [−0.27, −0.15] (*p* < 0.001) at 2–3 months, showing significantly decreased trait anxiety from baseline to both follow-ups. The following covariates were significant: baseline score and the interaction of time at 2–3 months and MEQ30. Therefore, trait anxiety scores reduced to a greater degree at the 2–3 month timepoint the higher the MEQ30 score.

#### Emotion regulation

**Figure 2:**
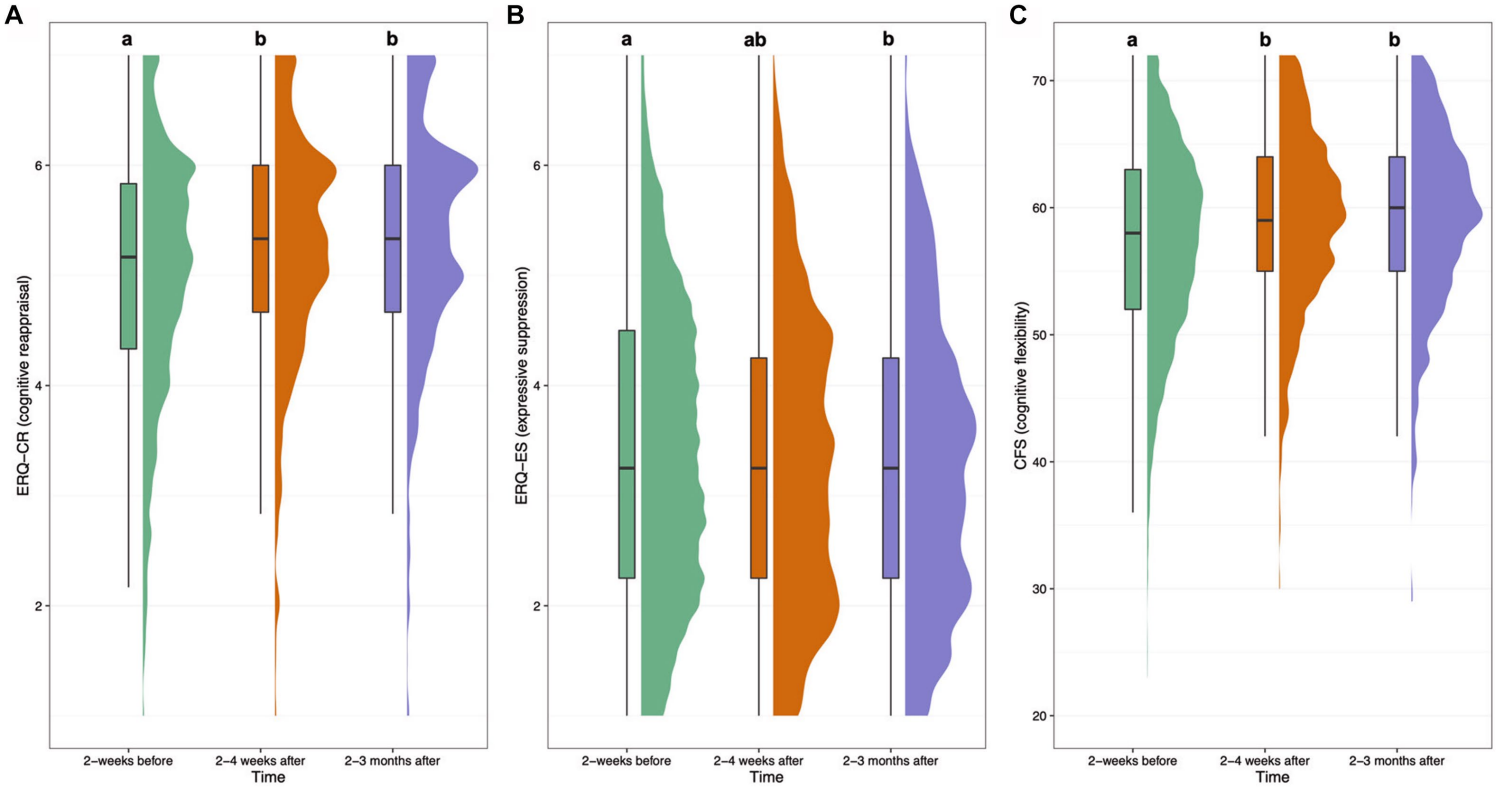
Boxplots and density plots for the following measures over time: Emotion Regulation Questionnaire (ERQ) cognitive reappraisal subscale **(A)**, ERQ expressive suppression subscale **(B)**, and Cognitive Flexibility Scale (CFS) score **(C)**. Boxes show the interquartile range of responses with the median at the solid horizontal line. Whiskers above and below boxes extend 1.5 times the interquartile range, and density plots show distribution of responses. Values not sharing a common letter above whiskers are significantly different (*p* < 0.0023). Graphs show plotted raw data from all participants with available data at each timepoint.

Mean (SD) ERQ cognitive reappraisal and expressive suppression scores on survey 2 were 5.0 (1.2) and 3.4 (1.3), respectively. For survey 5, ERQ cognitive reappraisal and expressive suppression scores were 5.2 (1.1) and 3.3 (1.3), respectively. For survey 6, ERQ cognitive reappraisal and expressive suppression scores were 5.3 (1.1) and 3.3 (1.3), respectively. Covariate-adjusted contrasts between each timepoint for ERQ cognitive reappraisal and expressive suppression scores are shown in Figure 2. The effect size (SMD [95% CI]) of ERQ-Cognitive Reappraisal at 2–4 weeks was 0.19 [0.11, 0.27] (*p* < 0.001), and 0.24 [0.16, 0.32] (*p* < 0.001) at 2–3 months, showing significantly increased cognitive reappraisal from baseline to both follow-ups. The following covariates were significant for cognitive reappraisal: baseline score, main effect of time at 2–4 weeks (positive), the interaction of time at the 2–3 month timepoint and MEQ30 (positive) and the interaction of time at both timepoints and TAS (negative). Therefore, cognitive reappraisal scores increased to a lesser degree over time in individuals who had higher TAS scores at baseline, and increased to a greater degree at the 2–3 month timepoint the higher the MEQ30 score. The effect size (SMD [95% CI]) of ERQ-Expressive Suppression at 2–4 weeks was −0.06 [−0.13, 0.01] (*p* = 0.11), and − 0.07 [−0.14, −0.01] (*p* = 0.03) at 2–3 months, showing no significant difference in expressive suppression from baseline to follow-ups. The following covariates were significant: baseline score and the interaction of time at the 2–4 week timepoint and TAS (positive). Therefore, expressive suppression was greater at 2–4 weeks the greater the TAS score at baseline.

#### Cognitive flexibility

Cognitive flexibility (CFS) mean (SD) total scores were 57.1 (7.8) for survey 2, 59.0 (7.0) for survey 5, and 59.1 (7.0) for survey 6. The effect size (SMD [95% CI]) of CFS at 2–4 weeks was 0.23 [0.15, 0.31] (*p* < 0.001), and 0.22 [0.15, 0.30] (*p* < 0.001) at 2–3 months, showing significantly increased self-reported cognitive flexibility from baseline to both follow-ups. The following covariates were significant: baseline score, and the interaction of time and MEQ30 at both timepoints (positive). Therefore, cognitive flexibility scores increased at a greater rate over time the higher the MEQ30 scores.

#### Spiritual wellbeing

**Figure 3:**
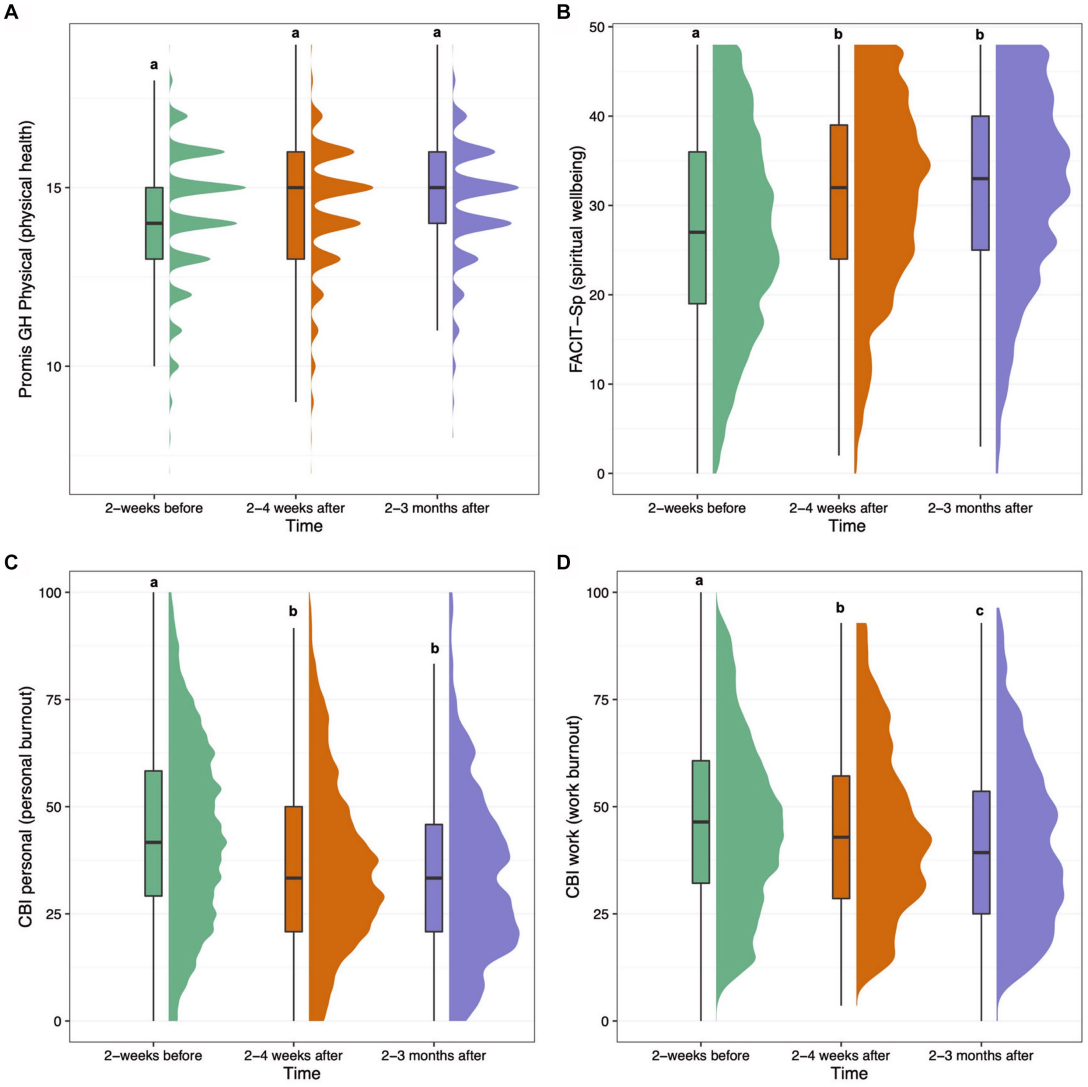
Boxplots and density plots for the following measures over time: PROMIS-GH Physical Health score **(A)**, FACIT-Sp Spiritual Wellbeing score **(B)**, Copenhagen Burnout Inventory (CBI) personal burnout score **(C)**, and CBI work burnout score **(D)**. Boxes show the interquartile range of responses with the median at the solid horizontal line. Whiskers above and below boxes extend 1.5 times the interquartile range, and density plots show distribution of responses. Values not sharing a common letter above whiskers are significantly different (*p* < 0.0023). Graphs show plotted raw data from all participants with available data at each timepoint.

FACIT-Sp spiritual wellbeing mean (SD) total scores were 27.1 (11.3) for survey 2, 31.0 (10.6) for survey 5, and 31.7 (10.5) for survey 6. The effect size (SMD [95% CI]) of FACIT-Sp at 2–4 weeks was 0.36 [0.29, 0.42] (*p* < 0.001), and 0.38 [0.31, 0.44] (*p* < 0.001) at 2–3 months, showing significantly increased spiritual wellbeing from baseline to both follow-ups. Covariate-adjusted contrasts between each timepoint for FACIT-Sp are shown in Figure 3. The following covariates were significant: baseline score, main effect of time at 2–3 months (positive), the interaction of time and MEQ30 at both timepoints (positive), State of Surrender (positive), the interaction of time and TAS at the 2–4 week timepoint (negative), and the interaction of time and ACE at the 2–4 week timepoint (positive). Therefore, spiritual wellbeing scores increased at a greater rate over time the higher the MEQ30 and ACE scores, while they increased at a lesser rate over time the greater the TAS scores, and greater State of Surrender was associated with greater spiritual wellbeing overall.

#### Burnout

Mean (SD) Copenhagen Burnout Inventory (CBI) personal and work-related burnout scores on survey 2 were 44.3 (21.4) and 42.2 (23.5), respectively. For survey 5, CBI personal and work-related burnout scores were 36.8 (19.8) and 39.4 (22.8), respectively. For survey 6, CBI personal and work-related burnout scores were 34.1 (20.0) and 35.6 (22.7), respectively. The effect size (SMD [95% CI]) of CBI Personal burnout at 2–4 weeks was −0.33 [−0.37, −0.29] (*p* < 0.001), and − 0.36 [−0.42, −0.31] (*p* < 0.001) at 2–3 months, showing significant decreases from baseline at both follow-up surveys. The following covariates were significant: baseline score, main effect of ACE (positive), the interaction of time and MEQ30 at both timepoints (negative), and the interaction of time and ACE at both timepoints (negative). This means personal burnout scores decreased at a greater rate over time the higher the MEQ30 scores, and that although higher ACE scores were associated with higher personal burnout across time, personal burnout decreased at a greater rate the higher the ACE score at baseline. The effect size (SMD [95% CI]) of CBI Work burnout at 2–4 weeks was −0.07 [−0.12, −0.01] (*p* = 0.01), and − 0.19 [−0.25, −0.12] (*p* < 0.001) at 2–3 months, showing significant decrease at the 2–3 month follow-up (survey 6), but not the 2–4 week follow-up (survey 5). The following covariates were significant: baseline score, and the interaction of time and MEQ30 at the 2–3 month timepoint (negative). Therefore, work burnout scores decreased at a greater rate at the 2–3 month timepoint the higher the MEQ30 scores.

#### Self-reported physical health

Mean (SD) PROMIS-GH Physical health scores for survey 2 were 14.1 (1.3), with mean (SD) scores of 14.4 (1.7) for survey 5, and 14.5 (1.6) for survey 6. The effect size (SMD [95% CI]) of PROMIS-GH Physical health at 2–4 weeks was −0.01 [−0.09, 0.07] (*p* = 0.99), and 0.06 [−0.02, 0.14] (*p* = 0.17) at 2–3 months, showing no significant change over time. Only the following covariate was significant: baseline score.

#### Personality

Mean (SD) extraversion significantly increased from 3.12 (0.86) at baseline to 3.17 (0.84) at 2–3 months (*p* < 0.001). Mean (SD) neuroticism significantly decreased from 2.88 (0.89) at baseline to 2.81 (0.87) at 2–3 months (*p* < 0.001). Mean (SD) agreeability increased non-significantly from 3.85 (0.6) at baseline to 3.87 (0.59) at 2–3 months, *p* = 0.11. Mean (SD) conscientiousness increased non-significantly from 3.64 (0.68) at baseline to 3.66 (0.67) at 2–3 months, *p* = 0.14. Mean (SD) openness was unchanged, from 4.11 (0.53) at baseline to 4.11 (0.52) at 2–3 months (*p* = 0.84).

#### Set and setting variables

Neither history of previous psychedelic use nor presence of a sitter/guide were significant predictors of outcomes in any of the longitudinal models. With regards to mindset, as stated above, greater State of Surrender before psilocybin use was associated with lower depression (BDI) overall and with smaller reductions in depression over time, but was not significantly associated with other outcomes. Greater absorption scores (TAS) at baseline were associated with decreased cognitive reappraisal (ERQ-Cr) and decreased spiritual wellbeing over time (FACIT-Sp), and increased expressive suppression (ERQ-Es) over time after psilocybin use. Additionally, insofar as long-term psychological impacts of adverse childhood experiences can be considered part of the set or setting of the experience, greater ACE scores at baseline were associated with higher levels of personal burnout (CBI Personal) overall, as well as greater decreases in depression (BDI) and personal burnout (CBI Personal), and greater increases over time in spiritual wellbeing (FACIT-Sp) after psilocybin use.

#### Attributions of meaning, spiritual significance, insight, and psychological challenge

At 2–4 weeks after the psilocybin experience, 353 (29.9%) respondents considered it to have been one of the 10 most personally meaningful of their lives; 333 (28.2%) considered it to have been one of the 10 most spiritually significant events of their lives; 370 (31.3%) considered it one of the 10 most psychologically insightful experiences of their lives; and 172 (14.6%) considered it one of the 10 most psychologically challenging experiences of their lives (Table 3). At 2–3 months after the psilocybin experience, 215 (32.7%) respondents considered it to have been one of the 10 most personally meaningful of their lives; 210 (32.0%) considered it to have been one of the 10 most spiritually significant events of their lives; 233 (35.5%) considered it one of the 10 most psychologically insightful experiences of their lives; and 109 (16.6%) considered it one of the 10 most psychologically challenging experiences of their lives.

#### Other behavioral changes after psilocybin use

Regarding other notable behavioral changes, positive changes were more commonly reported after psilocybin use as opposed to negative changes overall, and about 23% of individuals reported no notable behavioral changes at 2–4 weeks and 2–3 months after their psilocybin experience (Table 3). The most commonly reported behavioral changes after psilocybin use were improved relationships with others (*n* = 589; 49.8% at 2–4 weeks, and *n* = 332; 50.5% at 2–3 months), increased physical activity/exercise (*n* = 322; 27.2% at 2–4 weeks, and *n* = 212; 32.3% at 2–3 months), improvements in career/work life (*n* = 313; 26.5% at 2–4 weeks, and *n* = 208; 31.7% at 2–3 months), and improved diet/nutrition (*n* = 285; 24.1% at 2–4 weeks, and *n* = 182; 27.7% at 2–3 months). Overall, a large majority of respondents characterized their experience using psilocybin as beneficial 2–4 weeks and 2–3 months afterwards (92–94%) as opposed to rating it as neutral (5–7%) or negatively (1–2%). Regarding changes in wellbeing and life satisfaction associated with the psilocybin experience, at 2–4 weeks and 2–3 months, most respondents (84–89%) reported positive and desirable changes, 10–12% reported no change, and a few (1–4%) reported negative and undesirable changes.

### Relationships of personal history, personality, and mindset with subjective effects

#### Adverse childhood experiences

**Figure 4:**
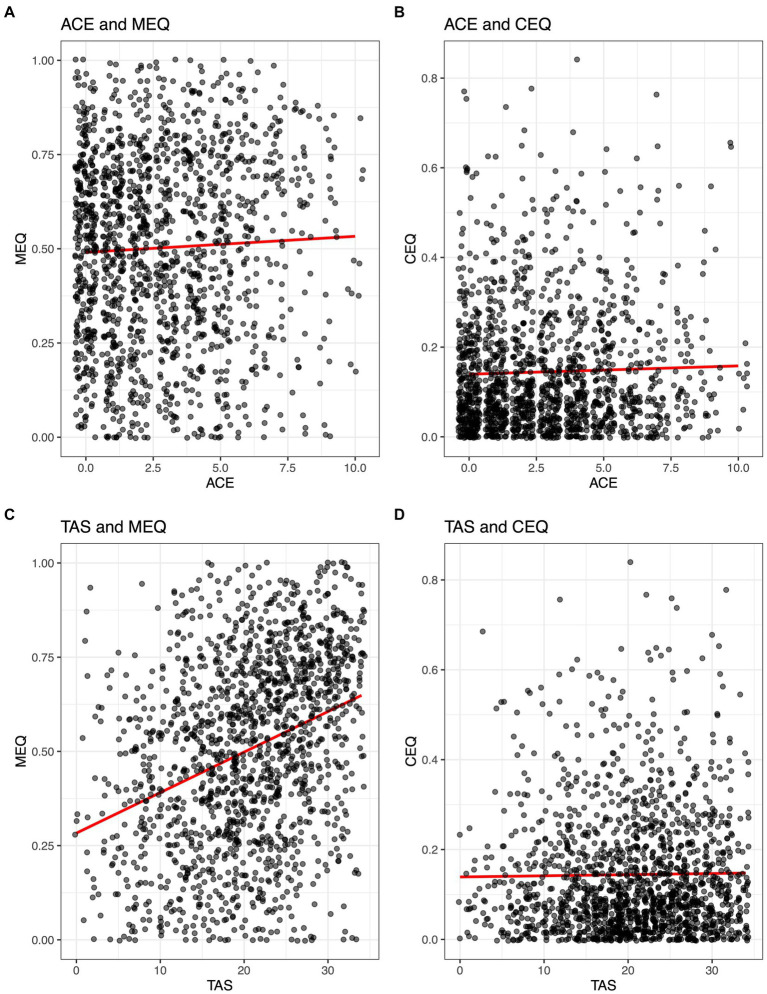
Correlations between Adverse Childhood Experiences (ACE) and Tellegen Absorption Scale (TAS) with mystical (MEQ30) and challenging (CEQ) subjective drug effects during psilocybin. Kendall correlation coefficients between ACE and MEQ30 scores were tau = 0.03 (*p* = 0.13; **A**), and for ACE and CEQ scores were tau = 0.02 (*p* = 0.4; **B**). Pearson correlation coefficients for TAS and MEQ30 were r = 0.32 (*p* < 0.001; **C**) and for TAS and CEQ were *r* = 0.01 (*p* = 0.58; **D**).

Adverse childhood experience (ACE) scores were not significantly associated with subjective drug effects (Figure 4). Kendall correlation coefficients between ACE and MEQ30 scores were tau = 0.03 (*p* = 0.1275), and for ACE and CEQ scores were tau = 0.02 (*p* = 0.4015), indicating no significant relationship between history of adverse childhood experiences and subjective drug effects.

#### Absorption

Tellegen Absorption Scale (TAS) scores were significantly associated with MEQ30 scores but not with CEQ scores. Pearson correlation coefficients for TAS and MEQ30 were *r* = 0.32 (*p* < 0.001) and for TAS and CEQ were r = 0.01 (*p* = 0.58), indicating a weak positive correlation between absorption and mystical-type effects, and no relationship between absorption and challenging drug effects (Figure 4).

#### State of surrender

**Figure 5:**
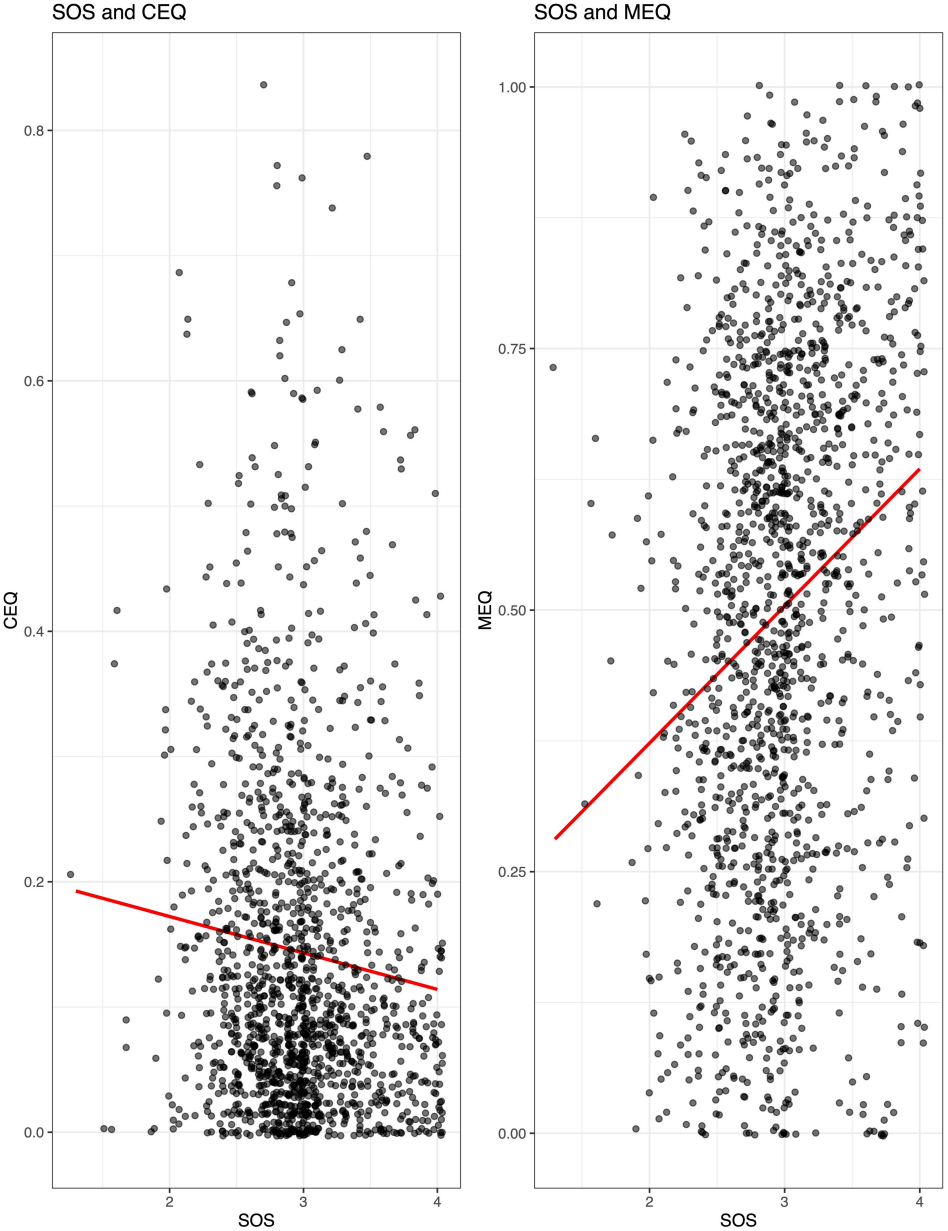
Correlations between State of Surrender (SOS) before psilocybin use and mystical (MEQ30) and challenging (CEQ) subjective drug effects during psilocybin. Pearson correlation coefficients for SOS and CEQ were *r* = −0.10 (*p* < 0.001; Left) and SOS and MEQ30 were *r* = 0.24 (*p* < 0.001; Right).

State of Surrender (SOS) scores were significantly associated with MEQ30 scores, and with CEQ scores. Pearson correlation coefficients for SOS and MEQ30 were *r* = 0.24 (*p* < 0.001) and for SOS and CEQ were *r* = −0.10 (*p* < 0.001), indicating a weak positive correlation between State of Surrender before session and mystical-type effects, as well as a very weak but statistically significant correlation between State of Surrender before session and challenging effects (Figure 5).

### Antidepressant use and acute subjective effects

The 72 participants who reported taking an antidepressant concurrently with psilocybin exhibited lower MEQ30 and CEQ scores than individuals who were not taking an antidepressant, indicative of weaker acute subjective effects among this subsample. For MEQ30, mean (SD) scores were 0.40 (0.26) for individuals using antidepressant medication compared to 0.51 (0.25) for those not taking an antidepressant (*p* = 0.0007). For CEQ, mean (SD) scores were 0.10 (0.13) for those individuals using antidepressant medication compared to 0.15 (0.14) for those not taking an antidepressant (*p* = 0.001).

## Discussion

This study presents the largest prospective, longitudinal dataset on naturalistic psilocybin use published to date. Similar to cross-sectional data on naturalistic psilocybin use (35), relatively few medically significant acute reactions (<4%) or persisting negative effects (<5%) were reported here. Nevertheless, some non-trivial issues were reported in a small minority of respondents, indicating a need for ongoing caution among both naturalistic users and clinical researchers to avoid negative outcomes such as mania or psychosis, which have been previously reported (35, 53). Positive behavioral changes were more frequently reported after psilocybin use than negative ones, with improved relationships being most commonly endorsed (~50% of respondents), and about 23% reporting no notable behavioral changes after their experience. In retrospect, most participants (>90%) viewed their naturalistic psilocybin use positively, and more than 80% attributed desirable changes in wellbeing and life satisfaction to their experience. Overall, data support prior evaluations of psilocybin as reasonably safe and non-toxic compared to other commonly used substances (54–56), with the caveat that individuals who experienced particularly difficult reactions or significant adverse events may not have been able or willing to respond to follow-up surveys as described in the study limitations.

Subjective effects data found low challenging experience scores on average and moderately high mystical experience scores, with roughly one fifth of the sample meeting *a priori* criteria for a “complete” mystical experience. Interestingly, acute challenging experiences were only weakly related to State of Surrender before the session and did not significantly predict any changes in longitudinal variables, suggesting that challenging experiences may pose acute risk but that they may not on average predict therapeutic outcomes. These data also indicate a need for better characterization of challenging psychedelic experiences and their potential relationship to persisting effects. Subjective effects ratings were lower and yielded a smaller proportion of “complete” mystical experience than those found in laboratory research administering 20 mg/70 kg or 30 mg/70 kg psilocybin (57). At follow-up surveys roughly 2 weeks and 2 months after the reference experience, between a quarter and a third of respondents considered the experience among the 10 most meaningful or spiritually significant of their lives, exhibiting lower rates of personal meaning and spiritual significance than both healthy (23, 58) and clinical populations (9, 18, 33) administered psilocybin in laboratory settings. However, the doses used in the present study are likely lower than those typically used in clinical research and previous studies have shown ratings of personal meaning, spiritual significance, and mystical-type effects of psilocybin (58), as well as related subjective effects such as oceanic boundlessness (59), to be dose dependent.

Longitudinal data indicate that among the convenience sample reported here, naturalistic use of psilocybin mushrooms was associated with significant improvements in mental health, wellbeing, and psychological functioning when controlling for demographic variables, in line with initial hypotheses. Persisting reductions in depression, state and trait anxiety, and alcohol misuse were found after psilocybin use, congruent with clinical studies showing similar results (9–12, 14, 17, 33, 60). Moreover, a moderately large effect size (SMD = 0.58–0.71) was observed for reductions in depression, providing additional support for psilocybin as a potential antidepressant treatment. However, it is notable that a substantial proportion of the current sample (>40%) met BDI-II criteria for depression at baseline and that the majority (>85%) had prior experience using psilocybin an average of 16–17 times before joining the study. These findings suggest psilocybin may have a time-limited window of antidepressant effects, consistent with clinical trial data (14, 15, 61), and imply that some individuals may require repeated psilocybin dosing for continued antidepressant benefits. Future research should examine methods for identifying such individuals, and the optimal interval and dosing regimen to be used in these cases.

In exploratory analyses, we found weaker acute subjective effects of psilocybin (i.e., mystical-type and challenging effects) among those concurrently taking antidepressants. This is consistent with similar survey findings of attenuated psychedelic response in individuals on antidepressants (62, 63). Although antidepressants were not differentiated by drug class in the present survey, it is likely the majority were serotonergic. Given the heterogeneous clinical presentation and small number of individuals taking antidepressants in the current study, impact of antidepressant use on enduring outcomes was not possible to adequately assess. However, it is possible that diminished subjective effects may not necessarily equate to reduced clinical efficacy in patient populations. Of note, a recent study found pre-treatment with the selective serotonin reuptake inhibitor (SSRI) escitalopram produced no difference in positive mood effects of psilocybin (25 mg) in healthy individuals (*n* = 23), but did elicit less bad drug effect, anxiety, and adverse effects compared to pre-treatment with placebo (64). Similarly, novel data showed 25 mg of psilocybin exhibited clinically meaningful antidepressant effects lasting up to 3 weeks in individuals with treatment resistant-depression (*n* = 19) who were concurrently maintained on an approved SSRI medication (65). Future targeted research on interactions between psilocybin and common psychiatric medications will be highly valuable to inform clinical practice (66, 67).

In addition to psychiatric constructs such as depression and anxiety, psilocybin also impacted broader areas related to emotion, cognition, wellbeing, and personality. Cognitive reappraisal, an aspect of emotional regulation defined as the ability to change one’s thoughts about emotionally charged stimuli, showed significant increases after psilocybin. However, expressive suppression, referring to inhibition of behavioral responses to emotionally charged stimuli, showed no change. These findings are the first to explicitly support psilocybin-related improvements in emotional regulation processes, in line with data suggesting that cognitive reappraisal strategies are generally associated with healthier patterns of social and emotional functioning than expressive suppression (68). Similarly, participants displayed significant increases in cognitive flexibility after psilocybin use, indicating greater awareness of alternative possibilities and willingness to adapt to a given situation. Prior studies have found self-reported cognitive flexibility to be negatively associated with mental health conditions such as anxiety and depression (69) and positively associated with constructs such as self-compassion (70). Cross-sectional survey studies have found that naturalistic psychedelic use may increase psychological flexibility, in turn mediating reductions in mental health symptoms such as anxiety and depression (13), as well as racial trauma among black, indigenous, and people of color (71). Similarly, the psychedelic ayahuasca has been found to increase cognitive flexibility and mindfulness in the period immediately following use (72), and psilocybin has also shown enhancement of cognitive flexibility in neuropsychological testing up to 4 weeks after drug administration in individuals with major depression (73). However, improvements in cognitive flexibility and reductions in depression were not directly correlated, suggesting an important, but nuanced role for cognitive flexibility in mediating mental health benefits of psychedelics (73).

Personal burnout showed significant reductions at both 2–4 weeks and 2–3 months after psilocybin use, and work-related burnout showed significant reductions at the 2–3 month follow-up only. To our knowledge, these are the first data showing psychedelic-associated reductions in burnout as a specific construct, which is characterized by fatigue and exhaustion in relation to high-stress work or living environments (46). No changes were found in self-reported physical health from before to after psilocybin use, suggesting minimal impact of naturalistic psilocybin use on physical health factors such as activities of daily living (e.g., walking), and providing a useful positive control for longitudinal data. However, significant and persisting increases in spiritual wellbeing after psilocybin use were observed in the present study, consistent with clinical trials administering high-dose psilocybin to cancer patients with anxiety and depressed mood (9, 11). Finally, regarding personality, only two dimensions showed significant changes after psilocybin use, with increases in extraversion and decreases in neuroticism observed, but no changes in openness, which has previously been reported to increase after psilocybin administration (74, 75).

Several hypotheses were tested in the current study. The first was borne out by longitudinal data showing general improvements across multiple domains of mental health and wellbeing. Regarding elements of mindset and their influence on subjective drug effects, consistent with previous findings, we found significant associations between absorption and State of Surrender before the psilocybin experience, and mystical-type effects during psilocybin use (31, 76). State of Surrender was weakly negatively correlated with challenging experience scores, and absorption showed no relationship to challenging experiences. History of adverse childhood experiences (ACE) was not significantly associated with either mystical or challenging subjective drug effects, contrary to initial hypotheses. However, greater baseline ACE scores were associated with decreased depression and personal burnout at follow-up and increased spiritual wellbeing at follow-up. Contrary to expectations, greater State of Surrender was not associated with outcomes other than lower depression overall and less decrease in depression at the 2–4 week timepoint. The latter finding could be related to regression toward the mean. Greater TAS was not associated with outcomes other than *decreased* cognitive reappraisal at both follow-up time points, decreased spiritual wellbeing at 2–4 weeks, and *increased* expressive suppression at 2–4 weeks. The origin and nature of these relationships remain somewhat unclear and may benefit from additional investigation.

Consistent with prior research, mystical-type effects were associated with persisting improvements in mood, anxiety, cognitive flexibility, and spiritual wellbeing. Additionally, novel findings presented here suggest mystical-type effects of psilocybin were associated with ongoing and significant reductions in personal burnout, an area that requires further study. Finally, presence of a sitter during psilocybin use was not associated with any persisting benefits, contrary to previous findings and our initial hypotheses (35), although such a finding does not preclude the idea that a sitter may reduce the likelihood of adverse events or risky behavior. Taken together, these findings indicate set and setting variables contribute strongly to perceived drug effects acutely as well as persisting effects, consistent with research showing notable placebo responses in carefully designed experimental environments (77, 78). In therapeutic settings, it is likely that these factors can be leveraged to enhance mental health outcomes over and above drug effects alone via additive and dynamic social processes such as psychological support and intentional meaning-making (21, 79, 80).

### Study limitations

The current study findings should be interpreted carefully in light of several limitations. Participant self-selection and homogeneity of the majority White and well-educated sample make it difficult to generalize findings to the wider population. Because the data were gathered anonymously online, it is also not possible to meaningfully verify participant responses. Response bias among this convenience sample may have influenced individuals to respond or to present themselves or their experience in a particular manner. In addition, response attrition in the current uncompensated, longitudinal survey study adds to concerns about generalizability and bias, as certain individuals may have been more or less likely to continue providing responses over time, potentially skewing results. Some participants also reported other concurrent substance use during their psilocybin experience, potentially confounding results. Furthermore, 26% of remaining participants at 2–4 week follow-up, and 50% of participants at 2–3 month follow-up had taken at least 1 subsequent dose of psilocybin, meaning the results here should also be considered in light of additional psilocybin use beyond the single reference experience.

The disproportionately White sample provides minimal data on underrepresented racial and ethnic minorities, congruent with current clinical research studies of psychedelics (81) and other drugs (82). While consistent with epidemiological data on naturalistic psychedelic use being primarily self-reported by White males (27), these results also perpetuate lack of knowledge and understanding regarding psychedelic use patterns and outcomes in non-White and other minoritized populations, representing an important area for future research to expand further (83). Race (i.e., White or non-White) was not a significant covariate related to any longitudinal outcomes. However, future analyses of the present dataset are planned to specifically investigate patterns and effects of naturalistic psilocybin use among non-White respondents.

## Conclusion

Despite the limitations noted above, findings from the present study are highly consistent with a growing body of clinical trial, behavioral pharmacology, and epidemiological data on psilocybin. Namely, these results indicate broad therapeutic potential of psilocybin to produce lasting improvements in mental health symptoms related to anxiety, depression, and substance misuse, and these benefits are often associated with subjective drug effects, including mystical-type experience. Additionally, psilocybin seems capable of producing enduring changes in psychological functioning and personality such as increased cognitive flexibility, emotion regulation, and extraversion, and reduced neuroticism, even in naturalistic settings that lack structured psychological support. Results suggest psilocybin holds further promise for spirituality and wellbeing enhancement in non-clinical populations, which warrants additional research (21, 25). However, consistent with previous reports, we found a small minority of individuals reporting harms from sessions or long-term negative effects. Overall, these data provide an important window into the current resurgence of public interest in classic psychedelics and the outcomes of contemporaneous increases in naturalistic psilocybin use. Though the findings reported here are generally positive in nature, questions remain about for whom such use may pose unnecessary risks, mechanisms underlying the persisting changes observed, and in what ways psilocybin’s unique profile of pharmacological effects may be optimally harnessed in clinical or other settings, presenting critical directions for future investigation.

## Data availability statement

The original contributions presented in the study are included in the article/supplementary material, further inquiries can be directed to the corresponding authors.

## Ethics statement

The studies involving humans were approved by the Johns Hopkins School of Medicine IRB. The studies were conducted in accordance with the local legislation and institutional requirements. The ethics committee/institutional review board waived the requirement of written informed consent for participation from the participants or the participants’ legal guardians/next of kin because no direct participant contact was involved for this online study.

## Author contributions

SN, HiJ, and NS made substantial contributions to the design of the study, the analysis and interpretation of the data, and the drafting of the manuscript. DM, SS, AY, HZ, TB, and ML made substantial contributions to participant recruitment, acquisition and interpretation of the data, and the drafting of the manuscript. DJ, HeJ, RG, and MJ made substantial contributions to the conception and design of the study, interpretation of the data, and made critical revisions to the manuscript. FB and JS made substantial contributions to the analysis and interpretation of the data and the drafting of the manuscript. AG-R made substantial contributions to the conception and design of the study, the analysis and interpretation of the data, and the drafting of the manuscript. All authors approved the final version of this manuscript and agree to be accountable for all aspects of the work.

## Funding

Funding for this research was provided by the Unlimited Sciences. The Center for Psychedelic and Consciousness Research was funded by philanthropic support from the Steven and Alexandra Cohen Foundation, Tim Ferriss, Matt Mullenweg, Blake Mycoskie, and Craig Nerenberg.

## Conflict of interest

FB was a scientific advisor for WavePaths, Ltd. and MindState Design Labs, Inc., and has provided consulting services for Gilgamesh Pharmaceuticals, Inc. RG was on the board of directors of the Heffter Research Institute. MJ has served as scientific advisor to AJNA Labs LLC, AWAKN Life Sciences Inc., Beckley Psychedelics Ltd., Entheogen Biomedical Corp., Field Trip Psychedelics Inc., Mind Medicine, Inc., Otsuka Pharmaceutical Development & Commercialization, Inc., and Negev Capital. AG-R was a paid scientific advisor to InnerWell Inc., NeonMind Biosciences, and ETHA Natural Botanicals.

The remaining authors declare that the research was conducted in the absence of any commercial or financial relationships that could be construed as a potential conflict of interest.

## Publisher’s note

All claims expressed in this article are solely those of the authors and do not necessarily represent those of their affiliated organizations, or those of the publisher, the editors and the reviewers. Any product that may be evaluated in this article, or claim that may be made by its manufacturer, is not guaranteed or endorsed by the publisher.
